# Cost-utility of triple versus dual inhaler therapy in moderate to severe asthma

**DOI:** 10.1186/s12890-021-01777-z

**Published:** 2021-12-05

**Authors:** Jefferson Antonio Buendía, Diana Guerrero Patiño

**Affiliations:** 1 Research Group in Pharmacology and Toxicology “INFARTO”, Department of Pharmacology and Toxicology, University of Antioquia, Medellín, Colombia; 2 Hospital Infantil Concejo de Medellín, Medellín, Colombia; 3 Facultad de Medicina, Universidad de Antioquia, Carrera 51D #62-29, Medellín, Colombia

**Keywords:** Tiotropium, Uncontrolled asthma, Cost-effectiveness analysis, Decision analysis, Markov model

## Abstract

**Background:**

An important proportion of asthma patients remain uncontrolled despite using inhaled corticosteroids and long-acting beta-agonists. Clinical guidelines recommend, in these patients, using add-on long-acting muscarinic antagonists (triple therapy) to treatment with high doses of inhaled corticosteroids-long-acting beta2-agonist (dual therapy). The purpose of this study was to assess the cost-effectiveness of triple therapy versus dual therapy for patients with severe asthma.

**Methods:**

A probabilistic Markov model was created to estimate the cost and quality-adjusted life-years (QALYs) of patients with severe asthma in Colombia. Total costs and QALYS of dual and triple therapy were calculated over a lifetime horizon. Multiple sensitivity analyses were conducted. Cost-effectiveness was evaluated at a willingness-to-pay value of $19,000.

**Results:**

The model suggests a potential gain of 1.55 QALYs per patient per year on triple therapy with respect to dual therapy. We observed a difference of US$304 in discounted cost per person-year on triple therapy with respect to dual therapy. The incremental cost-effectiveness ratio was US$196 in the probabilistic model. In the sensitivity analysis, our base‐case results were robust to variations in all assumptions and parameters.

**Conclusion:**

In conclusion, triple therapy in patients with moderate-severe asthma was cost-effective. Using triple therapy emerges with our results as an alternative before using oral corticosteroids or biologics, especially in resource-limited settings.

## Background

Asthma is the most prevalent respiratory disease in all age groups [1]. At least 24% of patients with asthma are classified as severe asthma requiring high doses of inhaled corticosteroids (ICS)-long-acting beta2-agonist (LABA) or ICS-LABA or oral corticosteroids (OCS) [2]. The direct cost of severe asthma per patient is three times higher than the cost of mild asthma; a cost that would be higher if we include indirect costs [3]. It is estimated that adults had 1.5 missed days per month due to asthma symptoms and 4.9 days per month of reduced productivity [4].In this sense, severe asthma is a serious problem for health systems. In US Yaghoubi and colleagues projected the economic and humanistic burden of asthma among U.S. adults from 2019 to 2038; they estimated that there will be around 175 million person-years with uncontrolled asthma and if all those people with uncontrolled asthma in the United States can achieve and maintain asthma control, the saving would be about $300 billion in direct costs and $660 billion in indirect costs, recovering 15,462 quality-adjusted life-years[5].

Gina 2021 recommends medium or high-dose ICS and LABAs combination ( dual therapy) as a preferred controller [6]. Indeed, despite these drugs, almost 70% of these patients do not achieve total control of symptoms [2]. Clinical guidelines recommend, in these patients, using add-on long-acting muscarinic antagonists (LAMA) to treatment with ICS-LABA in severe asthma because this triple therapy (ICS + LABA + LAMA) because improves lung function, quality of life and increased the time to severe exacerbation requiring OCS [6–9]. This is a relevant alternative, insofar as it can prevent the patient from ending up using oral corticosteroids or high-cost biologic drugs. However, this recommendation raises concerns as if the extra benefit offered by this drug outweighs the additional cost compared to therapy with only dual therapy. This question is even more relevant in developing countries with an increasing prevalence of asthma and constrained healthcare. An economic evaluation of these new drugs could provide evidence to optimize the efficiency of using economic resources in these countries. This study aimed to use to assess the health and economic consequences of dual inhaled therapy (LABA + ICS) versus triple inhaled therapy (LAMA + LABA + ICS) for the treatment of severe asthma in Colombia.

## Materials and methods

**Fig. 1:**
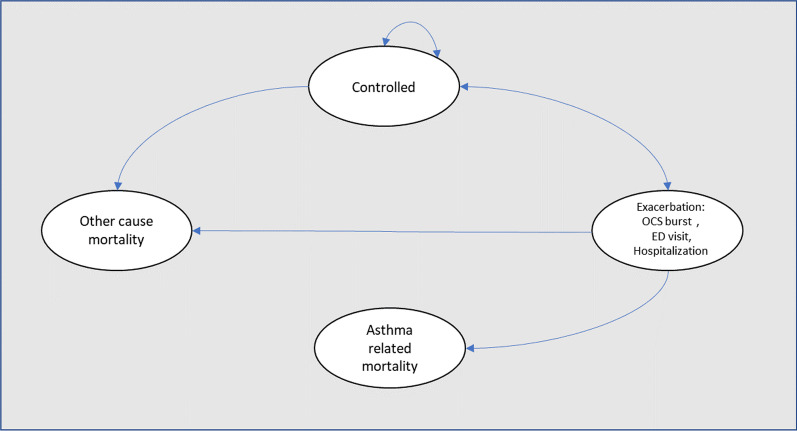
Markov model

We conducted a probabilistic Markov model to estimate the cost and quality-adjusted life-years (QALYs) of patients with severe asthma treated with dual inhaled therapy and triple inhaled therapy in Colombia. The choice of time horizon was a lifetime, using a cycle length of 2 weeks following the natural history of the disease and previously published asthma economic evaluation models [10–13]. In this mathematical model, patients could transition between four mutually exclusive health states (symptom-free state or asthma-controlled, asthma exacerbation, asthma-related mortality, and all-cause mortality). During each cycle, patients in non-death health states could transit to any of three levels of asthma exacerbations: OCS burst (was defined as relatively major symptoms during the week and need of use of oral corticosteroids to achieve the control of symptoms), emergency department (patient that request treatment with systemic corticosteroids) and hospitalization. Asthma-related mortality following an exacerbation or all-cause mortality could also occur (Fig. 1). We did this analysis from a societal perspective (including direct and indirect costs).. Half-cycle correction and an annual discounting rate of 5% were applied to both costs and QALYs, following the recommendations of the Colombian guide for health economic evaluations [14]. Treatment was considered cost-effective if the incremental cost-utility ratio was below $19,000 per QALY gained using the World Health Organization (WHO) recommendation of three times the GDP per capita to define the willingness to pay (WTP) in Colombia.

### Parameters of the Markov model

Multiple parameters were derived from published research and local data, which are presented in Table 1. Data of relative risk (RR) on exacerbation rates were extracted from a recent systematic review of dual versus triple therapy in patients with severe asthma [15]. In this study, triple therapy was associated with a reduction in severe exacerbation risk (9 trials [9932 patients]; 22.7% vs 27.4%; RR, 0.85 [95% CI, 0.77 to 0.90]). The transition probabilities for moving between different health states were derived from clinical trials and local studies [16, 17]. Data of utilities of each Markov state were extracted from a systematic review of utilities in asthma [18, 19], Table 1. This systematic review identifies 20 studies in asthma that report utilities in different severity states of asthma. Within these four studies (n = 330 patients) showed a median utility of 0.74 ± 0.029 for severe asthma, all estimated using a time trade-off or standard gamble or Asthma symptom utility index in the US and UK population. All these data (RR, transition probabilities, and utilities) were subjected to probabilistic sensitivity analysis as detailed below, and as recommended by Consolidated Health Economic Evaluation Reporting Standards (CHEERS) Statement [20]. In this sensitivity analysis, to build the range of RR to be used in this analysis, we use the CI 95% of RR published by clinical trials [15]. In the case of utilities and transition probabilities, the upper and lower ranges were estimated by adding or subtracting 25% of the value from the central value defined for the base case. The risk of asthma mortality and mortality from other causes was estimated using age- and gender-specific Colombian life tables mortality (2016 to 2020) [17, 21]. Based on previous studies of drug adherence, for dual and triple therapy, we assumed that 44% and 37% discontinued the treatment after 52 weeks of treatment respectively [22, 23]. Sensitivity analysis of percentage of non-adherents and response rates were made by estimating the upper and lower range of each value by adding or subtracting 25% of the value defined previously.

**Table 1:** Base case

Variable	Base case	Valor High	Valor Low	References
Cost Tiotropio (per 4 week cycle)	$ 60	$ 75	$ 45	[19, 23]
Cost Umeclidinium (per 4 week cycle)	$ 32	$ 40	$ 24	
Cost Glycopirronium (per 4 week cycle)	$ 32	$ 40	$ 24	
Cost ICS + LABA (per 4 week cycle)	$ 27	$ 34	$ 20	
Cost ED visit (per episode)	$ 26	$ 33	$ 20	
Cost hospitalization (per day)	$ 80	$ 100	$ 60	
Utilities (anual)				
Utility of controlled state	0.740	0.93	0.56	[13]
Utility decrement				
Exacerbations requiring OCS burst	0.1	0.13	0.08	[14]
Exacerbations requiring ED visit	0.15	0.19	0.11	
Exacerbations requiring hospitalization	0.2	0.25	0.15	
ICS + LABA + LAMA efect				
Relative risk on exacerbation rate	0.85	0.78	0.92	[10]
Adherence				
ICS + LAMA + LABA	63%	79%	47%	[17, 18]
ICS + LABA	56%	70%	42%	
Transition probabilities				
Probability controlled to OC Burst	0.12	0.12	0.07	[11]
Probability OCS Burst to ED visit	0.47	0.59	0.35	[12]
Probability of ED visti to hospitalization	0.1500	0.19	0.11	
Asthma mortality	0.00020	0.00024	0.00014	[16]
Annual dicount rate	5%	6%	0%	

All costs for each health state defined in the Markov model were extracted from a previously published Colombian-based study [24]. Briefly, this study identified the asthma-related direct and indirect costs of 1131 patients with severe asthma from January 1, 2004, through December 31, 2014, in Colombia, Table 1. Asthma severity classification was mainly based on the paper of Jacob et al. [25]. Severe persistent asthma required to have more than six Short-Acting Beta-Agonists (SABA) fills per year, and the number of OCS fills per year, was greater than or equal to two or 4 or more exacerbations. Moreover, zero to six SABA fills and three or more SABA fills per year also constitute severe asthma. This criterion related to using rescue medication per year may be more accurate than using LABA + ICS given the high frequency of underuse and prescription of controller medications in Latin American countries [26]. This group of patients with severe asthma had an average of 1.4 ED visits per year, and 2.5 hospitalizations per year; rates that are comparable to those reported in clinical trials and observational studies in patients with severe asthma and tiotropium use [7, 27]. Drug’s cost and drug’s share market of dual (included Budesonide/Formoterol 640/18 mcg daily, Fluticasone/Vilanterol 200/15 mcg daily, Fluticasone/Salmeterol 550/50 mcg daily) and triple therapy (included umeclidinium 50 mcg daily, glycopyrronium 20 mcg daily and tiotropium 5 mcg daily) was taken from the National Drug Price Information System [28]. All cost costs were transformed to 2020 costs using official inflation data in Colombia. We used US dollars (Currency rate: US$1.00 = COP$ 3,500) to express all costs in the study [21].

### Sensitivity analysis

To explore parameter uncertainty of the model inputs, first, we conducted a deterministic sensitivity analysis using one-way sensitivity analysis with their tornado diagrams, respectively. In this analysis, we univariate evaluated the change in the incremental cost-effectiveness ratio by varying each parameter as described above. Also to explore parameter uncertainty of the model inputs, we conducted a probabilistic sensitivity analysis by randomly sampling from each of the parameter distributions (beta distribution in the case of relative risk and utilities, Dirichlet distribution for multinomial data in the case of transition probabilities, and gamma distribution in the case of costs). The expected costs and expected QALYs for each treatment strategy were calculated using that combination of parameter values in the model. This process was replicated one thousand times (i.e., second-order Monte Carlo simulation) for each treatment option resulting in the expected cost-utility. All analyses were done in Microsoft Excel®.

## Results

Base-case analyses showed that triple therapy was associated with higher costs and QALYs than dual therapy. The model suggests a potential gain of 1.55 QALYs per patient per year on triple therapy with respect to dual therapy. In the analysis of the Markov cohort model, we estimated a median probability of surviving free of exacerbation of 0.87 and 0.85 for triple and dual therapy, respectively. We observed a difference of US$304 in total discounted cost per person-year on triple therapy concerning dual therapy, Table 2. The incremental cost-effectiveness ratio was US$196 in the probabilistic model and US$589 in the deterministic model.

**Table 2:** Cost- effectiveness of triple versus dual therapy

	Cost (US$)	Difference (US$)	QUALYs	Difference	C/E (US$)	ICER(US$)
Triple Therapy	416	304	7.1	1.5	58	196
Dual Therapy	111		5.6		20	

### Sensitivity analysis

**Fig. 2:**
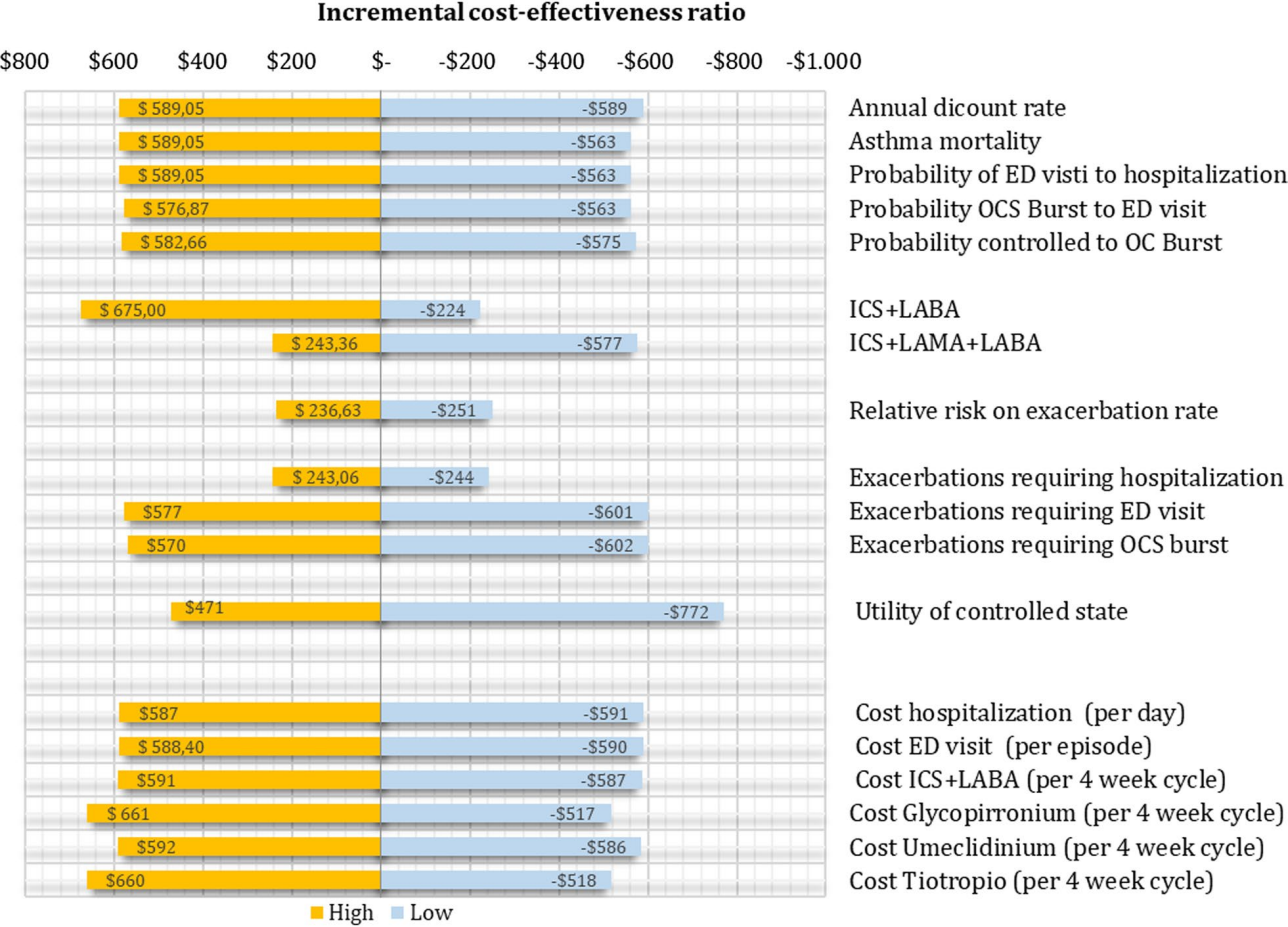
Tornado diagram

**Fig. 3:**
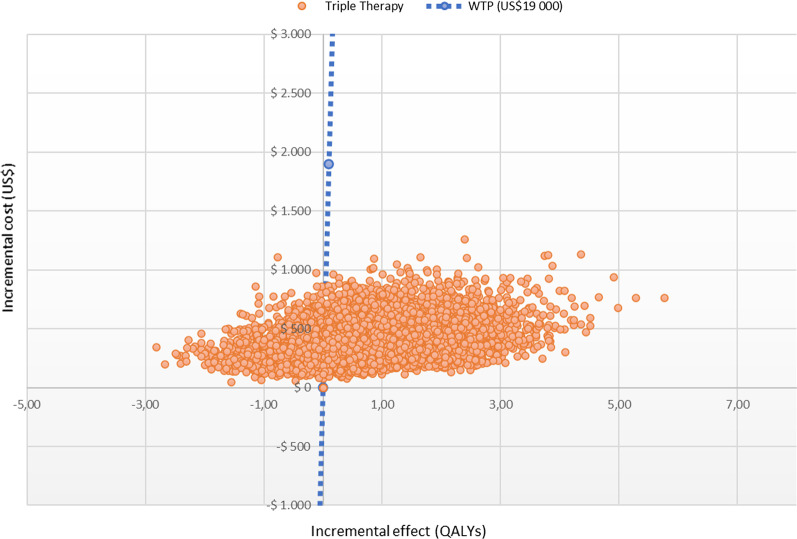
Cost effectiveness plane

**Fig. 4:**
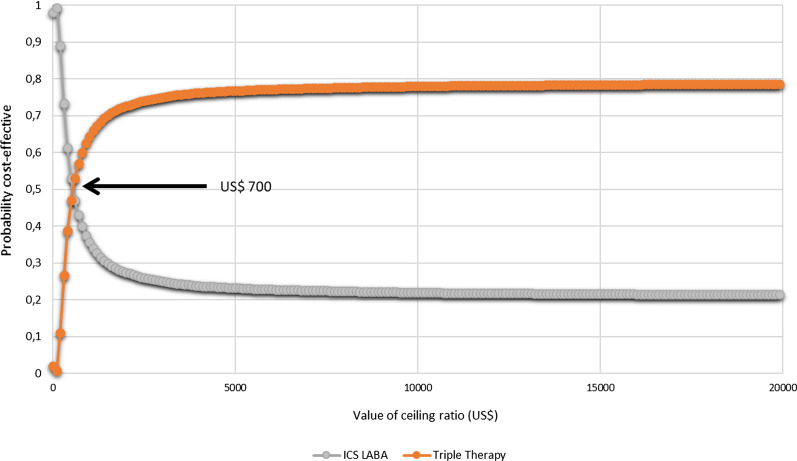
Cost-effectiveness acceptability curve

In the deterministic sensitivity analysis, our base‐case results were robust to variations in all assumptions and parameters. For none of the variables evaluated, variations within the established ranges led to the incremental cost-effectiveness ratio being higher than the WTP, Fig. 2. The results of the probabilistic sensitivity analysis are graphically represented in the cost-effectiveness plane, Fig. 3. This scatter plot shows that compared with dual therapy, treatment with triple therapy tends to be associated with lower costs and higher QALY. Indeed, 80% in quadrant 1 (high cost, high QALYs) and 20% in quadrant 4 (high cost, lower QALYs). The cost-effectiveness acceptability curve shows that triple therapy becomes cost-effective after willingness-to-pay thresholds of US$700; Fig. 4.

## Discussion

This study showed that triple was cost-effective than dual therapy in adolescent and adult patients with moderate-severe persistent asthma. Our findings support the GINA 2021 recommendations for using add-on LAMA to treatment with ICS-LABA asthma as an alternative in patients in Step 5. As we show this option generates 1.5 quality-adjusted life-year extra per patient concerning dual therapy with a cost of US$197, below of willingness to pay US 19 000 per QALY in Colombia. Using triple therapy emerges with our results as an alternative before using oral corticosteroids or biologics, especially in resource-limited settings.

Our results are in line with previous studies. Hyng et al., using a similar Markov model as our study, found, that in patients with poorly controlled asthma the adding tiotropium to ICS/LABA is a cost-effective alternative with an ICER $4,078/QALY in frequent SABA users and $8,332/QALY, on frequent exacerbators [27]. The differences in the magnitude of ICER are due to differences in the healthcare systems of Colombia and Korea and medical expenses. Indeed, our costs per event of OCR bust, ED visit, or hospitalization were 69%, 79%, and 46% less, respectively than in Korea. Wilson et al. using a six Markov model health states, estimate an incremental cost-effectiveness ratio of £21,906 per QALY gained being adding tiotropium to ICS/LABA cost-effective in the UK[29]. As is expected, the cost per event of an OCS bust, ED visit, or hospitalization was five times higher in the UK than in our study in Colombia; This can explain the differences in the magnitude of ICER between the studies. Zafari et al., using also a probabilistic Markov model with a 10-year time horizon and from a US societal perspective, found ICER of add-on therapy with tiotropium versus standard therapy, and omalizumab versus tiotropium was $34,478/QALY, and $593,643/QALY, respectively [30]. Despite differences in the model health states, higher costs of drugs and other direct costs in the US, and utilities, our conclusion is the same. One difference in our study to previous studies was the values of the utilities. The two previous studies used the utilities established in the Wilson study, which estimated them in the "PrimoTinAasthmatrial" population using the EuroQol EQ-5D tool in the UK population. We decided to use those reported in a systematic review to have broader values and in more diverse populations. Variations in the values of these utilities in the probabilistic sensitivity analysis did not significantly change the calculated ICER. Indeed, after of 10 000 simulations in our PSA tiotropium tends to be associated with lower costs and higher QALY; 80% of simulations were graphed in quadrant 1 of cost-effectiveness plane.

A not minor difference in our evaluation from previous studies is the fact that we have not only estimated the ranges of relative risks and transition probabilities using data from real-life studies but have adjusted our estimates for drug adherence. Assuming 100% adherence is unrealistic and tends to overestimate the effect of dual or triple therapy. A crucial methodological aspect is discussing willingness to pay (WTP) to declare Colombia a cost-effective technology or not. Since Colombia does not have a threshold that represents the WTP per unit of effectiveness (QALY), the ICER results per QALY were evaluated by using the reference corresponding to the World Health Organization (WHO) recommendation (three times the GDP per capita). Not having an own estimate of the WTP may be debatable; however, up to now, all the economic evaluations in health carried out in the country follow the threshold suggested by the WHO, which has also been endorsed by the national technology evaluation agency [31]. The results of the probabilistic sensitivity analyses confirm the robustness of the model results. Since relative risk and some transition probabilities and utilities do not come from the Colombian population, they were subjected to probabilistic sensitivity analysis as detailed below as recommended by Consolidated Health Economic Evaluation Reporting Standards (CHEERS) Statement[20].

Our study has some limitations. We use utilities extracted from the literature and not estimated directly from our population. As was mentioned previously, the reliability and robustness of the results were evaluated by sensitivity analysis. Our results only refer to patients with severe asthma uncontrolled by medium-dosage to high-dose inhaled corticosteroids plus long-acting β_2_-agonists and cannot be extrapolated to patients with using oral daily corticosteroids. Studies of triple therapy have recruited both allergic and non-allergic asthma patients. By using evidence from such trials, we assumed the same health benefits of tiotropium for allergic and non-allergic asthma patients, and this assumption is supported by trials of tiotropium, which showed no difference between allergic versus non-allergic subjects [7].

In conclusion, triple therapy in patients with moderate-severe asthma was cost-effective. Triple therapy emerges with our results as an alternative before using oral corticosteroids or biologics, especially in resource-limited settings.

## Data Availability

BD dual versus triple therapy asthma [Data set]. Zenodo. http://doi.org/10.5281/zenodo.5068083.

## Abbreviations

ICS: Inhaled corticosteroids
LABA: Long-acting beta2-agonist
OCS: Oral corticosteroids
LAMA: Long-acting muscarinic antagonists
QALYs: Quality-adjusted life-years
SOC: Standard therapy
WTP: Willingness to pay
RR: Relative risk
CHEERS: Consolidated Health Economic Evaluation Reporting Standards
SABA: Short-Acting Beta-Agonists
ICER: Incremental cost-effectiveness ratio
